# Impact of the Acuros XB spatial discretization error on ArcCHECK VMAT QA for small‐field SBRT

**DOI:** 10.1002/acm2.14100

**Published:** 2023-08-10

**Authors:** Chuan He, Ankit Pant, Anh H. Le

**Affiliations:** ^1^ Roswell Park Comprehensive Cancer Center Buffalo New York USA; ^2^ University at Buffalo The State University of New York Buffalo New York USA; ^3^ Department of Radiation Oncology Cedars‐Sinai Medical Center Los Angeles California USA

**Keywords:** Acuros XB, ArcCHECK, spatial discretization error, VMAT QA

## Abstract

**Purpose:**

To evaluate the impact of the Acuros XB spatial discretization errors on ArcCHECK volumetric modulated arc therapy (VMAT) QA for small‐field SBRT plans.

**Methods:**

Eighteen SBRT VMAT arcs that failed the ArcCHECK VMAT QA were retrospectively analyzed. Plan verification doses were calculated using Eclipse Acuros XB, and absolute 3%/2 mm gamma passing rates were calculated to compare ArcCHECK and MapCHECK2 with MapPHAN. Verification doses were recalculated using AAA in Eclipse and with the EGSnrc Monte Carlo package. In addition, error‐reduced Acuros XB doses were calculated by subdividing the entire arc into several sub‐arcs (“split‐arc” method), with the angular ranges of the sub‐arcs optimized to balance accuracy and efficiency. Relative gamma passing rates were calculated and compared for the four methods: (1) Acuros XB; (2) AAA; (3) EGSnrc Monte Carlo; and (4) the split‐arc method.

**Results:**

The absolute gamma passing rates were below 90% for ArcCHECK and above 95% for MapCHECK2. The averaged relative gamma passing rates were (1) 84.7% for clinical Acuros XB; (2) 96.8% for AAA; (3) 98.8% for EGSnrc Monte Carlo; and (4) 96.8% for the split‐arc method with 60° sub‐arc angle. Compared to the clinical Acuros XB, the split‐arc method improved the relative gamma passing rate by 12.1% on average. No significant difference was found between AAA and the split‐arc method (*p* > 0.05).

**Conclusion:**

The Acuros XB spatial discretization errors can significantly impact the ArcCHECK VMAT QA results for small‐field SBRT plans. The split‐arc method may be used to improve the VMAT QA results.

## 1 INTRODUCTION

External beam radiation therapy (EBRT) techniques improved over time from traditional conformal static fields to modern dynamic delivery methods like intensity‐modulated radiation therapy (IMRT), and volumetric modulated arc therapy (VMAT). These modern techniques can achieve better target coverage and normal tissue sparing with increased plan complexity due to simultaneous variations of gantry speed, dose rate, and MLC leaf motions. To ensure that treatments can be delivered as intended, measurement‐based patient‐specific VMAT QA is recommended by AAPM TG 218,^1^ and can be accomplished with a QA device to measure the delivered dose and an accurate dose calculation algorithm. One such device is the ArcCHECK (Sun Nuclear, Melbourne, FL), a 3D PMMA cylindrical diode array that can detect VMAT delivery errors.^2^, ^3^, ^4^ Gamma analysis is commonly performed during the VMAT QA to compare measured and calculated dose distributions. Gamma passing rate (GPR) is the percentage of the compared dose points that pass the selected gamma criterion (e.g., 3%/2 mm).

For the Eclipse Treatment Planning System (Varian Medical Systems, Palo Alto, CA), the Analytical Anisotropic Algorithm (AAA) and Acuros XB (AXB) are commonly used for photon beam dose calculation. In the literature, most studies have focused on the dosimetric comparison between AXB and AAA in high‐dose regions for general plans with relatively large field sizes, and AXB was commonly reported to be more accurate for heterogeneous tissues.^5^, ^6^, ^7^, ^8^ However, their results were mainly influenced by the heterogeneity effect in high‐dose regions and other impacts were overlooked for low doses, especially for highly modulated small‐field plans, which could impact the VMAT QA results. For example, AXB uses discretized resolution in space to boost the computational speed that causes discretization errors. The AXB computational grid size is not a user‐adjustable parameter and does not take a constant value throughout the calculation volume. It is internally determined and uses a finer resolution for the high dose region (over 15% isodose line for long arc VMAT) and a coarser resolution for the peripheral low dose region with reduced dose calculation accuracy.^9^ This impacts the VMAT QA results using ArcCHECK which measures dose in the peripheral low‐dose region and can have a disproportionate impact in small‐field cases, particularly for SBRT which delivers high doses at the central region with sharp dose falloff at the periphery of the target.

This study aims to evaluate the impact of the AXB spatial discretization errors on ArcCHECK VMAT QA GPRs for small‐field SBRT plans. A simple method is introduced to reduce this effect and improve the VMAT QA results.

## 2 METHODS

### 2.1 Clinical AXB data collection

Eighteen SBRT VMAT arcs that failed the VMAT QA have been retrospectively analyzed with 3.5 × 3.5 cm^2^ field sizes: nine arcs for lung SBRT plans with 6MV‐FFF energy and nine arcs for pelvic node plans with 10MV‐FFF energy. All arcs were calculated with the Eclipse AXB algorithm reporting dose‐to‐medium and delivered on a TrueBeam LINAC (Varian Medical Systems, Palo Alto, CA) with HD120 MLC. VMAT QA was performed with true composite measurement methodology recommended by TG‐218 using ArcCHECK and MapCHECK2 with MapPHAN. The clinical verification dose was calculated in Eclipse using the AXB algorithm with a 1.25 mm dose grid size for each arc. Gamma analysis was performed using SNC Patient software (Sun Nuclear Corporation, Melbourne, FL). The absolute GPRs were calculated with the recommended criteria from TG‐218: 3%/2 mm, 10% threshold, global normalization, and action limit of 90%.

### 2.2 Secondary AAA and Monte Carlo dose calculation

To compare AXB with other algorithms, verification doses were recalculated with AAA in Eclipse and Monte Carlo (MC) previously modeled for TrueBeam LINAC using the EGSnrc toolkit.^10^ Different ArcCHECK virtual phantoms were used for these algorithms with different assigned vendor‐recommended densities, including 217 HU scaled water for AAA and 1.1836 g/cm^3^ PMMA for MC (Table 1). To eliminate the dose scaling differences caused by daily machine output changes and various ArcCHECK virtual phantom densities, relative GPRs were calculated for comparison.

**TABLE 1:** Specifications of ArcCHECK virtual phantoms.

Algorithms	Specifications	Comments
AXB	AXB PMMA	AXB PMMA material assigned in Eclipse
AAA	217 HU scaled water	Default HU of the vendor‐provided virtual CT
MC	1.1836 g/cm^3^ PMMA	Default vendor‐recommended physical density

### 2.3 AXB using the split‐arc method

**FIGURE 1:**
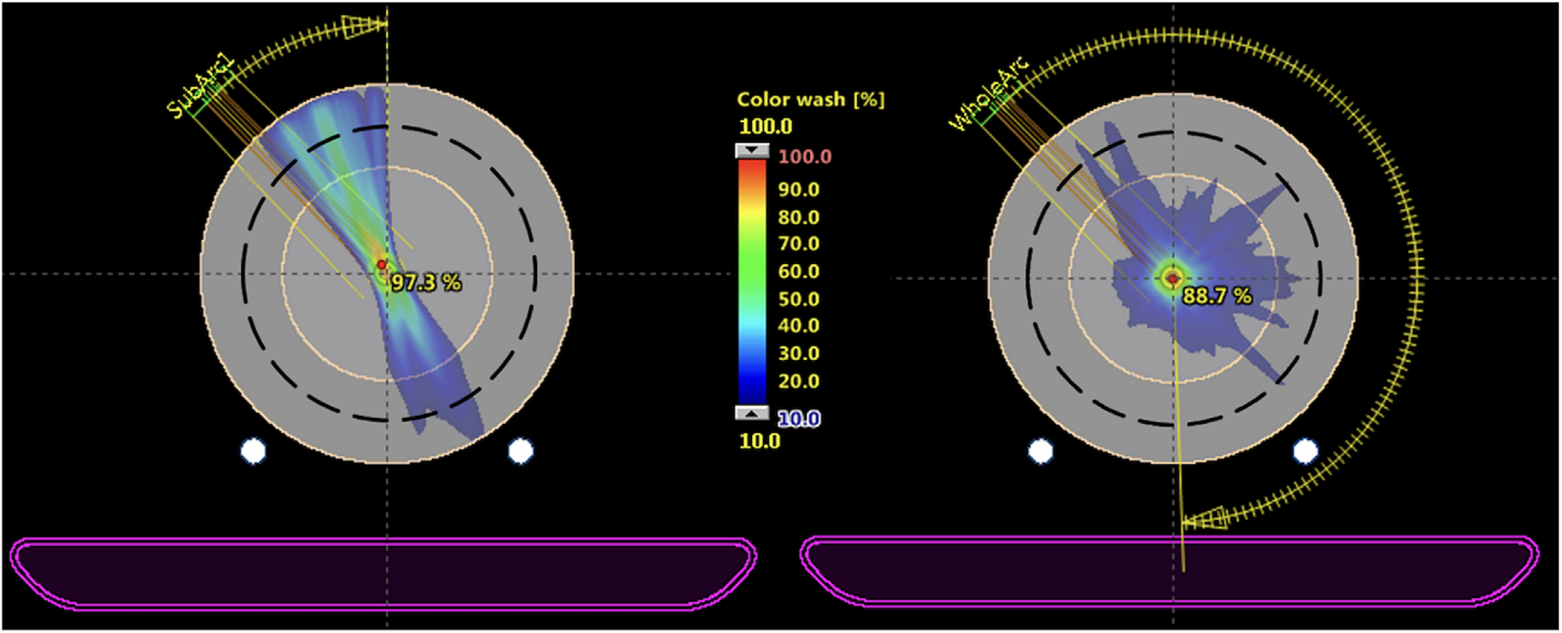
Example of relative dose distributions for a short arc field (left), and a long arc field (right). Compared to the long arc field that delivers high doses at the isocenter, the short arc field has higher doses distributed in the peripheral region that contains ArcCHECK detectors shown as the black dash‐line.

**FIGURE 2:**
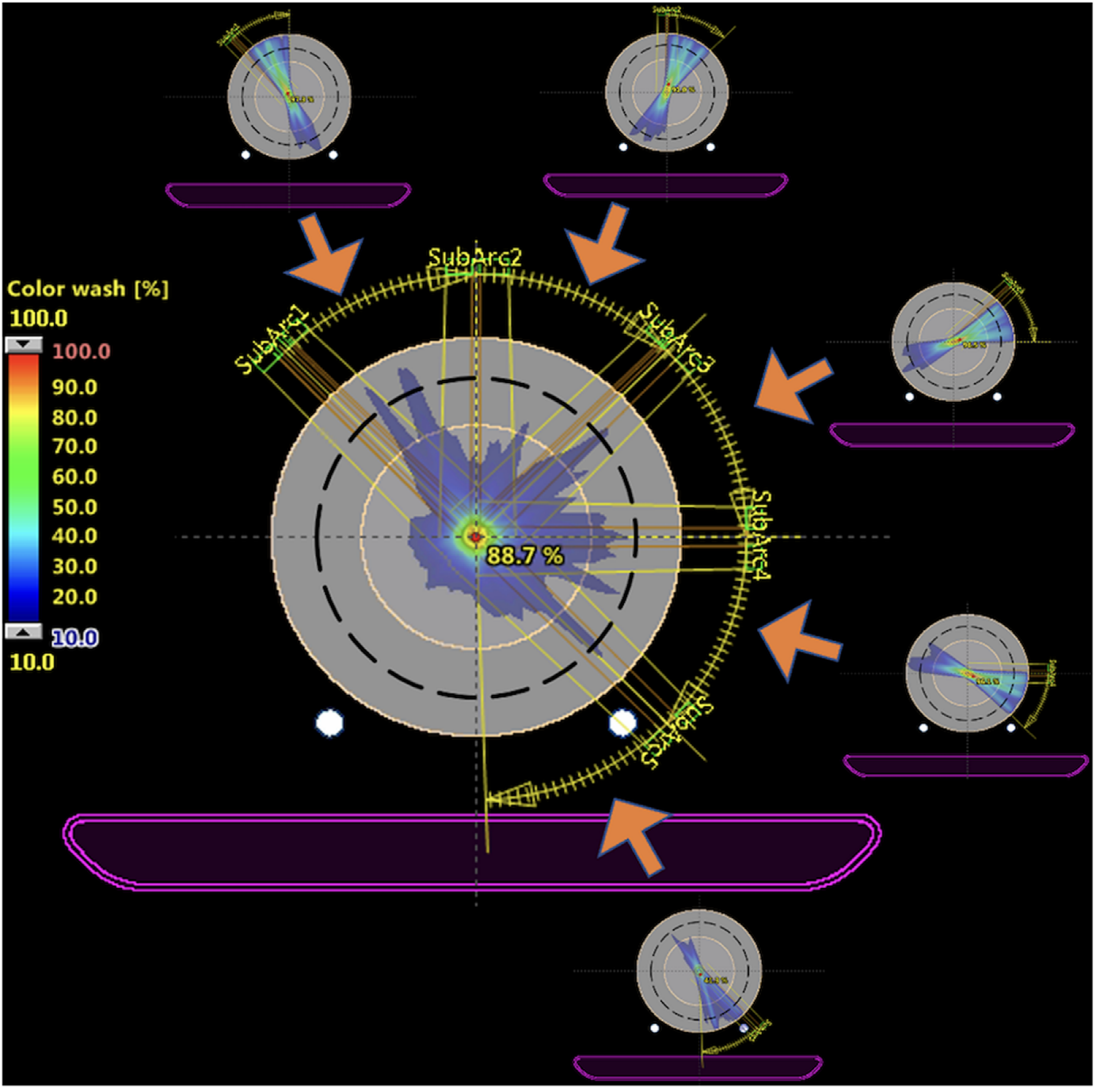
Example of the error‐reduced AXB dose using the split‐arc method. The entire arc is first split into five sub‐arcs, the dose is calculated for each sub‐arc, and the dose from all sub‐arcs is combined to yield the composite dose.

The split‐arc method was introduced to reduce the effect of the AXB spatial discretization error on ArcCHECK VMAT QA. Instead of directly calculating the dose to the entire arc as the clinical method, each arc field was first split into multiple sub‐arcs using a standard option when creating arc verification plans in Eclipse; the AXB dose was then calculated for each sub‐arc, and these partial doses were combined to yield the composite dose. The rationale is that compared to a long arc field with inaccurate low peripheral doses calculated, a short arc field contains high peripheral doses with better accuracy where the ArcCHECK detectors are located (examples shown in Figures 1 and 2). To find the optimal sub‐arc angle as a balance between accuracy and time efficiency, relative GPRs were calculated with 45°, 60°, and 90° sub‐arc angles, and the related calculation time was recorded. The optimal sub‐arc angle was chosen with an averaged GPR like AAA and the shortest average calculation time. Doses were calculated using the split‐arc method with the selected optimal sub‐arc angle, and the relative GPRs were calculated for comparison.

### 2.4 Comparisons of clinical AXB versus the split‐arc method and different algorithms

To quantitatively evaluate the impact of the AXB spatial discretization error on VMAT QA, relative GPRs were compared between the clinical AXB and the (1) AAA, (2) MC, and (3) error‐reduced AXB with the split‐arc method. Based on the comparison, recommendations were provided on handling the AXB spatial discretization error and improving the VMAT QA results for small‐field SBRT plans.

## 3 RESULTS

### 3.1 Absolute GPRs for clinical AXB

**FIGURE 3:**
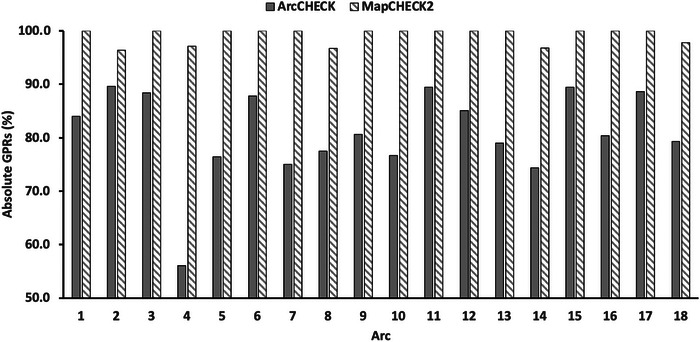
Absolute GPRs for the selected 18 arcs. The VMAT QA results for MapCHECK2 are acceptable (>95%), while for ArcCHECK are all below the action limit (<90%).

Figure 3 shows the clinical AXB absolute GPRs for the selected 18 arcs for ArcCHECK and MapCHECK2. Although all ArcCHECK VMAT QA failed, the absolute GPRs were all above 95% for MapCHECK2.

### 3.2 Accuracy and time efficiency for the split‐arc method

**FIGURE 4:**
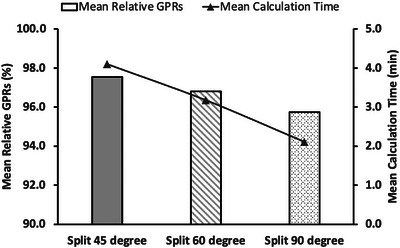
Mean relative GPRs and mean calculation time for different sub‐arc angles using the split‐arc method. For a smaller sub‐arc angle, the mean GPR is higher, but the mean calculation time is longer.

Figure 4 shows the relative GPR and calculation time on average for different sub‐arc angles using the split‐arc method. The mean GPRs for 45°, 60°, and 90° were 97.5%, 96.8%, and 95.7%. Although the smaller sub‐arc angle had a better GPR on average, the mean calculation time also increased (4.1 min for 45°, 3.2 min for 60°, and 2.1 min for 90°).

### 3.3 Quantitative comparison using relative GPRs

**FIGURE 5:**
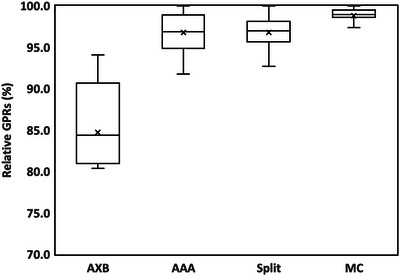
Comparison of relative GPRs among clinical AXB, AAA, split‐arc method with 60° sub‐arc angle, and MC. The boxplot shows the minimum, first quartile, median, mean showed as cross symbol, third quartile and maximum. Compared to the clinical AXB, the split‐arc method has a 12.1% better average result, like AAA (*p* > 0.05).

As shown in Figure 5, the averaged relative GPRs were 84.7% for clinical AXB, 96.8% for AAA, 96.8% for split‐arc with a 60° sub‐arc angle, and 98.8% for MC. The clinical AXB had the worst result, and the MC was the best. By reducing the AXB spatial discretization error using the split‐arc method, the result got 12.1% higher on average than clinical AXB. AAA and the split‐arc method had the same mean relative GPR and no significant difference was found (*p* > 0.05).

## 4 DISCUSSION

Unlike QA devices with 2D detector planes that measure the central planar dose close to the isocenter, ArcCHECK has a helical detector arrangement measuring the peripheral dose 10.4 cm away from the isocenter. For most of the general VMAT cases with large field sizes, this unique geometry is optimal for detecting minor delivery errors with advanced analysis like the gantry angle virtual inclinometer and control point beam eye‐view (BEV) dose analysis. However, for small‐field SBRT AXB plans with rapid dose fall‐off, while the ArcCHECK can measure low peripheral doses accurately, the VMAT QA results are still poor because of the reduced calculation accuracy due to AXB spatial discretization errors. Thus, the ArcCHECK VMAT QA results are impacted by the AXB spatial discretization error, reducing detection sensitivity for other delivery errors. As shown in Figure 3, all MapCHECK2 GPRs were above 95%, indicating these arcs were deliverable. However, the ArcCHECK GPR on average was 18.2% lower, which was misleading and time‐consuming for troubleshooting and re‐planning. Thus, the ArcCHECK detector geometry is not optimal for small‐field SBRT VMAT QA. Another QA device that measures the central high doses surrounding the isocenter may be preferable, like SRS MapCHECK designed explicitly for small‐field SRS plans. If ArcCHECK is the only choice due to the financial situation, it is critical to minimize errors due to Acuros XB discretization to improve ArcCHECK VMAT QA results. To our knowledge, there are currently no studies or vendor recommendations addressing this issue. This work is the first to quantitatively evaluate the impact of the AXB spatial discretization error on ArcCHECK VMAT QA and introduce the split‐arc method as a simple solution.

Since relevant parameters of the Eclipse AXB algorithm are not user‐customizable, it is not possible to directly increase the accuracy by changing the AXB parameters. Instead, the split‐arc method is a simple way to “trick” the AXB algorithm by creating sub‐arc fields with higher relative doses distributed at peripheral regions. The field split function is a standard option when making arc verification plans in Eclipse, so neither additional script nor technical support is necessary. As shown in Figures 4 and  5, the 60° sub‐arc angle had the same GPR on average as AAA (96.8%), and a smaller angle had a better GPR with a longer calculation time. From these results, a sub‐arc angle smaller than 60 degrees is recommended to achieve a better result than AAA.

Since the split‐arc method induces additional steps and calculation time, another well‐commissioned dose calculation algorithm may be used instead when efficiency is favored. As shown in Figure 5, MC had the best overall result, and AAA performed similarly to the split‐arc method with a sub‐arc angle of 60 degrees (*p* > 0.05). Unlike MC requiring additional computing resources, AAA can be directly used in Eclipse. Thus, AAA is recommended over MC to achieve acceptable time efficiency and sufficient precision as the split‐arc method with a sub‐arc angle of 60 degrees.

As a limitation, this study did not evaluate the impact of the AXB spatial discretization error on patient plan doses, especially for peripheral organ‐at‐risk (OAR). Future study is needed to investigate this impact and give recommendations for the secondary dose check on patient plan dose. The split‐arc method may also help reduce the AXB spatial discretization error, increasing the calculation accuracy of the patient dose.

## 5 CONCLUSION

This study demonstrates that the AXB spatial discretization errors can significantly impact the ArcCHECK VMAT QA GPRs for small‐field SBRT plans. Methods need to be implemented to minimize this impact and improve the VMAT QA results. Our recommendations are summarized as follows: (1) The ArcCHECK detector geometry is not optimal for small‐field SBRT VMAT QA. Other devices are recommended to measure the central high‐dose regions directly; (2) If the ArcCHECK is the only choice, the split‐arc method may be used to reduce the impact of the AXB spatial discretization error. A sub‐arc angle smaller than 60 degrees is recommended to achieve better accuracy than AAA; and (3) if available, the AAA algorithm may be reasonably used when time efficiency is favored over accuracy.

## AUTHOR CONTRIBUTIONS

Conception or design of the work: Chuan He, Ankit Pant, Anh H. Le. Data collection: Chuan He, Ankit Pant. Data analysis and interpretation: Chuan He. Drafting: Chuan He, Anh H. Le. Critical revision of the draft: Anh H. Le. Final approval of the version to be published: Anh H. Le.

## CONFLICT OF INTEREST STATEMENT

The authors declare no conflicts of interest.
